# On the problem of inflation in transcriptome-wide association studies

**DOI:** 10.1101/2023.10.17.562831

**Published:** 2023-10-20

**Authors:** Yanyu Liang, Festus Nyasimi, Hae Kyung Im

**Affiliations:** 1 Section of Genetic Medicine, University of Chicago, Chicago, Illinois, United States of America; 2 Computing Environment and Life Sciences Directorate, Argonne National Laboratory, Argonne, Illinois, United States of America

## Abstract

Hundreds of thousands of loci have been associated with complex traits via genome-wide association studies (GWAS), but an understanding of the mechanistic connection between GWAS loci and disease remains elusive. Genetic predictors of molecular traits are useful for identifying the mediating roles of molecular traits and prioritizing actionable targets for intervention, as demonstrated in transcriptome-wide association studies (TWAS) and related studies. Given the widespread polygenicity of complex traits, it is imperative to understand the effect of polygenicity on the validity of these mediator-trait association tests. We found that for highly polygenic target traits, the standard test based on linear regression is inflated $\left(E\chi_{\text{twas}}^{2}>1\right)$. This inflation has implications for all TWAS and related methods where the complex trait can be highly polygenic—even if the mediating trait is sparse. We derive an asymptotic expression of the inflation, estimate the inflation for gene expression, metabolites, and brain image derived features, and propose a solution to correct the inflation.

## Introduction

To explain the mechanism behind the hundreds of thousands of loci discovered via genome-wide association studies (GWAS), researchers have studied the role of molecular traits as mediators, leading to development of transcriptome-wide association studies (TWAS) and related methods for other molecular traits (Gamazon et al., 2015; Gusev et al., 2016; Zhang et al., 2022). These mediator-trait association studies have been increasingly important in genetic studies. However, a recent paper (Leeuw et al., 2023) has reported statistical inflation in TWAS. This inflation could be falsely identifying loci as putatively causal; therefore, it is important that we identify the source of the inflation error and correct for it.

Leeuw et al. (2023) attributed the observed inflation to the prediction error in the mediator. However, established error-in-variable literature (Fuller, 1987) indicates error in the independent variable does not cause inflation. The Leeuw et al. (2023) derivation conflates the true parameter (an unknown but fixed number) and the estimated parameter (a random variable). The proposed null hypothesis sets the estimated regression coefficient to be zero, which is an event of probability zero. This is naturally not a reasonable null hypothesis.

We show in this study that the inflation in TWAS and related methods is actually due to the polygenicity of the target trait. Most complex traits are highly polygenic (Yang et al., 2010; O’Connor et al., 2019; Boyle et al., 2017), and while the effect of polygenicity has been explored and leveraged in the context of GWAS with methods such as linkage disequilibrium (**LD**) score regression and related approaches (Bulik-Sullivan et al., 2015), the effect of polygenicity on TWAS has not been explored rigorously.

Here, we demonstrate analytically that when the target trait has a polygenic component, the distribution of the $\chi_{\text{twas}}^{2}$ statistics of the associations between the target trait and the gene expression (or other mediators) under the null has a positive noncentrality parameter, rather than the standard $\chi^{2}$ with a mean = 1, causing the observed inflation. Further, we characterize the properties of this inflation, highlight that prediction error does not cause inflation, show the effect of prediction error in the power of the test, demonstrate the practical relevance of inflation in real TWAS, and, finally, propose a solution to correct it.

## Results

TWAS and related methods nominate potential causal mediators (gene expression, protein levels, etc.) by testing the effect of the mediating trait T on a target trait Y assuming the following model:

$$Y=\beta \;T+\epsilon_{twas}$$

$$T=\sum_{k}\;\gamma_{k}\;X_{k}$$

where β is the (fixed) effect of the mediator on the target trait, $\epsilon_{twas}$ is the component unexplained by the mediator and is independent of it. $\gamma_{k}$ are the genetic effects on the mediator and $X_{k}$ are the genotype dosages. $\gamma_{k}$ and $\epsilon_{\text{twas}}$ are normally distributed and independent of each other. To allow for sparse architecture of the mediator, $\gamma_{k}$ can have a positive probability of being 0. The normality assumption can be replaced by finite variance assumption for all variables. However, traditional TWAS ignores the effect polygenicity of the target trait and prediction error may have on the association statistics.

To examine the effect of the polygenicity of the target trait Y on the association statistics, we explicitly modeled the direct genetic effects $\delta_{k}$ on the target trait using equation (1). To account for prediction error, we took into account that the TWAS regression is performed against a noisy version of the mediator $\tilde{T}$.

(1) $$Y=\beta \;T+\sum_{k}X_{k}\;\delta_{k}+\epsilon$$

(2) $$T=\sum_{k}\gamma_{k}\;X_{k}$$

(3) $$\tilde{T}=\sum_{k}\left(\gamma_{k}+e_{\gamma ,k}\right)X_{k}$$

where $\delta_{k}$, $\gamma_{k}$, $e_{\gamma ,k}$, and ϵ are all normally distributed and independent of each other. As in traditional TWAS, $\gamma_{k}$ can have a positive probability of being 0 to include sparse architecture for the mediator. For simplicity and without loss of generality, we assumed that Y, T, and $X_{k}$ have mean = 0 and variance = 1.

To quantify the effect of polygenicity and prediction error, we examined the asymptotic distribution of the $\chi^{2}$ statistic of the regression of the phenotype Y on the noisy predicted expression $\tilde{T}$. We call this statistic $\chi_{\text{twas}}^{2}$ to emphasize that this is the statistic that the standard TWAS method would calculate when the polygenicity and prediction error are present but ignored. As shown in the Methods section, the $\chi_{\text{twas}}^{2}$ statistic has a noncentral $\chi^{2}$ distribution with the mean given by

(4) $$E\chi_{twas}^{2}\approx 1+N{\;h}_{\delta }^{2}\frac{\Phi }{1\;-\;\beta^{2}\tau^{2}}+N\frac{\beta^{2}\tau^{2}}{1\;-\;\beta^{2}\tau^{2}},$$

where N is the sample size, β is the effect of the mediator T on the target trait Y, and $h_{\delta }^{2}$ is the polygenic portion of Y, i.e., the heritability of the target trait explained by the genetic effects $\delta_{k}.\;\Phi$ is defined as:

(5) $$\Phi =\frac{1}{M}\frac{{\tilde{\gamma }}^{\prime }\;\cdot \;\Sigma^{2}\;\cdot \;\tilde{\gamma }}{{\tilde{\gamma }}^{\prime }\;\cdot \;\Sigma \;\cdot \;\tilde{\gamma }},$$

where M is the number of causal SNPs for the target trait, Σ is the limit of R for N→∞, and $\tilde{\gamma }=\gamma +e_{\gamma }$ is the M-dimensional vector of noisy prediction weights. $\tau^{2}$ is the precision of the prediction of the mediator, i.e., the signal to noise ratio of T:

(6) $$\tau^{2}=\frac{var(T)}{var(\tilde{T})}.$$

The precision $\tau^{2}$ is also known as the reliability ratio in the error-in-variables literature (Fuller, 1987).

### Polygenicity of target trait Y causes inflation under the null

Under the null, $\beta^{2}=0$ so that the expected $\chi^{2}$ in equation (4) reduces to:

(7) $$E\chi_{twas}^{2}\approx 1+N{\;h}_{\delta }^{2}\;\Phi .$$

**Figure 1:**
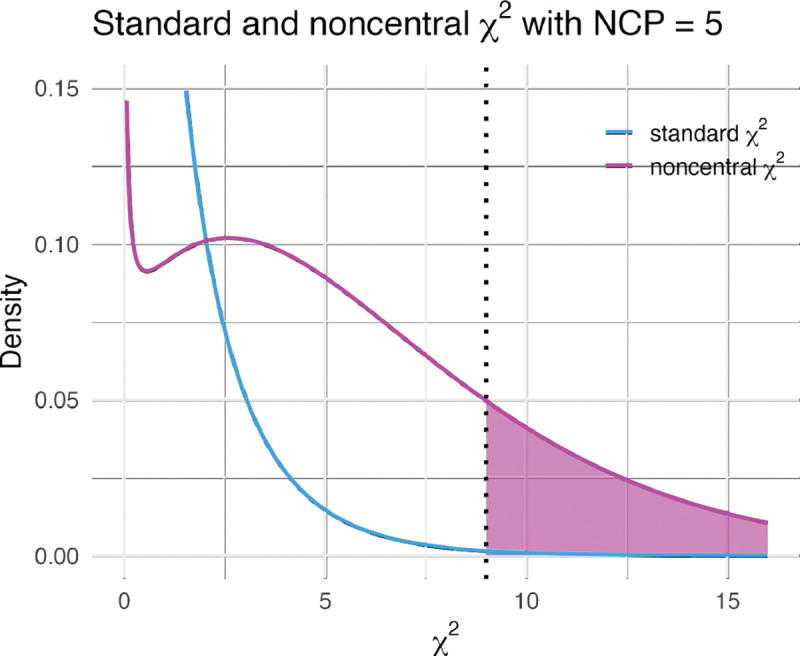
Example of standard and noncentral $\chi^{2}$ distributions. The probability densities of a standard and noncentral $\chi^{2}$ with noncentrality parameter (NCP)=5 are shown. We would get a noncentrality parameter of 5 under realistic parameters such as $\Phi =2\;\cdot \;10^{-5}$, N=500,000, and $h_{\delta }^{2}=0.50$. For an observed $\chi^{2}=9$ a standard $\chi^{2}$ would yield $p=2.6\;\cdot \;10^{-3}$ (shaded area under the blue curve to the right of the threshold), whereas with the noncentral $\chi^{2}$ would yield p=0.22 (shaded area under the pink curve), illustrating the miscalibration induced by the polygenicity of the target trait.

One immediate implication of this result is that the standard TWAS test—which assumes that $E\chi^{2}=1$ when in fact $E\chi^{2}=1+Nh_{\delta }^{2}\;\Phi$—will yield inflated p-values. The degree of inflation is illustrated in Figure 1 using an example. As discussed below, we will correct this inflation by estimating Φ and using the correct null distribution (i.e., the noncentral $\chi^{2}$ with noncentrality parameter ${Nh}_{\delta }^{2}\Phi$) to calculate the p-values of the association.

### Characterization of the inflation and definition of the mediator specific factor Φ

The inflation in TWAS is determined by the noncentrality parameter, which under the null is $Nh_{\delta }^{2}\Phi$. This term is positive when the target trait is polygenic, i.e. it has nonzero $h_{\delta }^{2}$. It is linear in $h_{\delta }^{2}$ and N. The dependence on the number and LD of the causal SNPs is encapsulated in the factor Φ.

For better understanding of how Φ behaves, we investigated its properties. From the definition in equation (5), we can see that Φ is only a function of the number of causal SNPs of the target trait M, the prediction weights $\tilde{\gamma }$, and the LD matrix Σ. which is the large N limit of $X^{\prime }\;\cdot \;X/N$ and thus is no longer dependent on N. Therefore, it does not depend on the sample size, heritability, or other properties of the target trait. This indicates that Φ is a property of the mediator, which can be pre-calculated and applied to any target trait Y.

We also show that Φ is strictly positive, bounded below and above as follows:

(8) $$\frac{1}{M}\leq \Phi \leq 1,$$

The lower bound is attained when Σ is the identity matrix, and the upper bound is achieved when the SNPs are perfectly correlated (Methods). When the SNPs are independent, we have:

$$\Phi =\frac{1}{M}\;\;\;\text{(independent}\;\text{SNPs)}$$

and hence the $E\chi_{\text{twas}}^{2}\approx 1+\frac{N}{M}{\;h}_{\delta }^{2}$ under the null.

The factor Φ is independent of the precision of the predictor $\tau^{2}:\tau^{2}$ is a scalar that affects both the numerator and denominator of Φ through the noisy weights $\tilde{\gamma }$ and therefore has no net effect. This result is more evident when we assume polygenicity of the mediator trait, in which case, Φ is given by:

(9) $$\Phi =\frac{tr\left(\Sigma^{2}\right)}{M^{2}}\;\;\text{(polygenic}\;\text{mediator).}$$

### Prediction error has no effect on the inflation under the null

**Figure 2:**
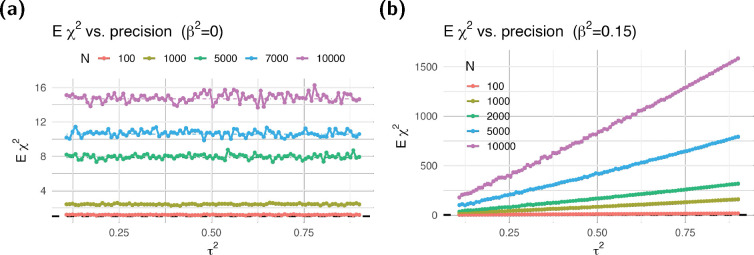
Dependence of $\chi^{2}\;statistics\;on\;the\;precision\;\tau^{2}$ of the prediction under the null and alternative. We calculated expected association $\chi^{2}$ statistics from simulations with different heritability values $h_{\delta }^{2}$ of the target trait, number of causal SNPs M, and sample sizes N. We took the average over 1,000 simulations, as well as over $h_{\delta }^{2}$ and M. Dash-dotted lines indicate the predicted $\chi^{2}$ statistics from equation (7) under the null and equation (4) under the alternative.

Next, we examined the effect of the precision of the prediction of the mediator on the inflation. Under the null, both T and estimated $\tilde{T}$ are independent of the target trait and have exactly the same dependence on genotype data; thus, the expected $\chi^{2}$ should not change. Indeed, we corroborated this by verifying that, under the null, the simulated expected $\chi^{2}$ is constant across all values of the precision as shown in Figure 2a.

Under the alternative, we find that $E\chi_{twas}^{2}$ increases monotonically with the precision of the prediction as shown in Figure 2b, indicating that prediction error reduces the power of the test. Details of the simulation are described in the following section.

Consistent with established literature (Fuller, 1987), our results show that prediction error in the mediator causes a loss of power, but it does not affect the inflation under the null—contrary to the conclusion in Leeuw et al. (2023).

### Validation of inflation equation under the null

To assess how well this approximation works under the null, we simulated both the target trait Y and the mediating trait T from infinitesimal models with independent effect sizes according to the equations (1) to (3). We simulated genotype data using independent binomial random variables with probability of 0.4—corresponding to the minor allele frequency of the SNPs—and therefore assumed no LD between SNPs. We used a range of values for the heritability of $Y\;\left(h_{\delta }^{2}\;:0.1-0.9\right)$, sample sizes (N:100-10,000), and number of causal SNPs (M:99-6000).

For each combination of $h_{\delta }^{2}$, N, and M, we simulated 1000 target traits Y and 99 mediating traits Ts and $\tilde{T}s$ unrelated to Y. Each of the 99 predicted mediating traits were simulated with different levels of precision $\left(\tau^{2}\;:0.10-0.9\right)$. We then regressed each target trait on each mediating trait separately and averaged the square of the Z-scores across the 1,000 simulations, thereby obtaining an estimated $E\chi_{twas}^{2}$ for each combination of $h_{\delta }^{2}$, N, M, $T_{k}$, and $\tau^{2}$.

**Figure 3:**
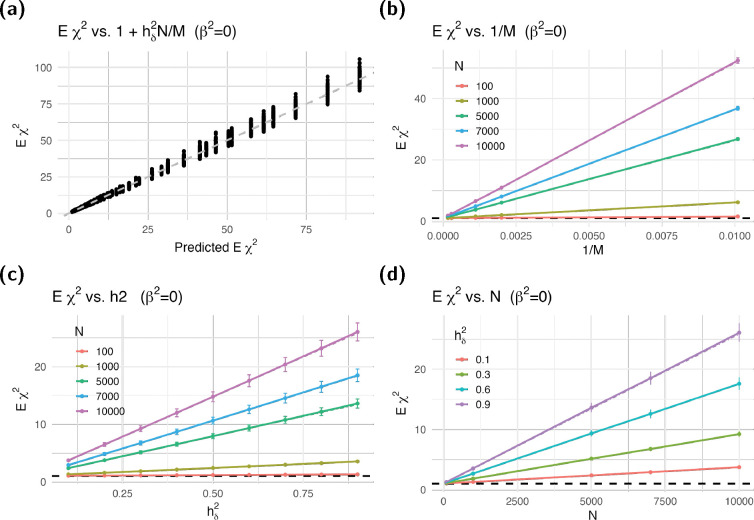
Expected $\chi^{2}$ vs. heritability, sample size, and inverse of the number of causal SNPs. We calculated the expected association $\chi^{2}$ statistics from simulations with different number of causal SNPs M, heritability values $h_{\delta }^{2}$, and sample sizes N. We took the average over 1,000 simulations and 99 independent mediators T. Panel (**a**) shows the average $\chi^{2}$ vs $1+h_{\delta }^{2}N/M$. Panels (**b-d**) show the average $\chi^{2}$ against 1/M, $h_{\delta }^{2}$, and N. The horizontal dashed line at 1 indicates where a calibrated $\chi^{2}$ statistics should be. The error bars represent the 95% confidence intervals from the simulations. Dash-dotted lines in the figure show the predicted $\chi^{2}$ statistics from equation (7) under the null—the expected $\chi^{2}$ mostly obscures the dash-dotted line, indicating the linear relationship is consistent with the theoretical approximation; We used M-1 for M, which further improved the match.

This estimated value was well approximated by our theoretical expression under the independent SNP assumption as shown in Figure 3a, where data points fall in the vicinity of the identity line. Panels b–d of the figure corroborate the linear relationship between the expected $\chi^{2}$ and 1/M, $h_{\delta }^{2}$, and N, respectively, as predicted by our equation (7).

### Validation of inflation equation under the alternative

To examine the effect of the prediction error on the test under the alternative, we used the same simulation setup we used for the null hypothesis with $\beta^{2}>0$. Hence, we simulated the target trait $Y=\beta \;T+\sum_{k}\;\delta_{k}\;X_{k}+\epsilon$ and the mediating trait $T=\sum_{k}\;\gamma_{k}\;X_{k}$. We performed the association using the noisy version of the mediator, ${\tilde{T}}_{k}=\sum_{k}\;\tilde{\gamma_{k}}\;X_{k}$.

**Figure 4:**
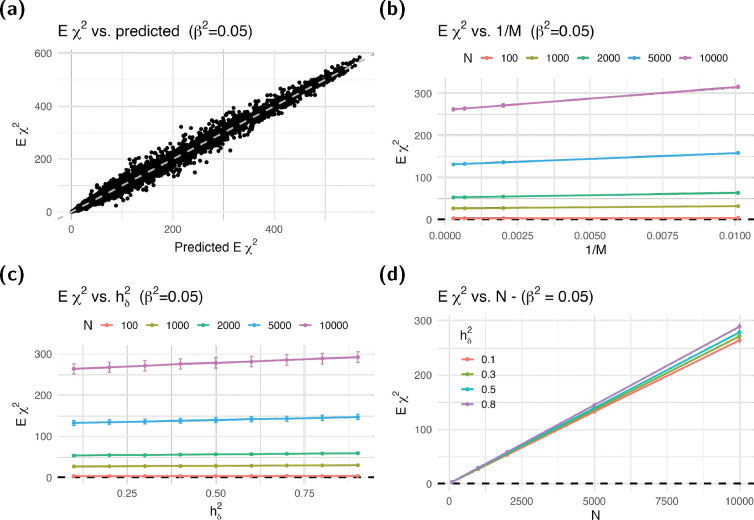
Expected $\chi^{2}$ under the alternative. We calculated expected $\chi^{2}$ statistics by averaging 1000 simulations of target trait Y with different combinations of number of causal SNPs M, polygenic portion of the heritability of $Y{\;h}_{\delta }^{2}$, and sample sizes N. Panel (**a**) shows the expected $\chi^{2}$ against the theoretically predicted value in equation (4) assuming independent SNPs. Dashed gray line shows the identity line. Panel (**b-d**) show the expected $\chi^{2}$ against 1/M, $h_{\delta }^{2}$, and N. Dash-dotted lines in the figure show the predicted $\chi^{2}$ statistics from equation (4) assuming independent SNPs—for most conditions, the match is good enough to obscure the dash-dotted line in the figure. We used M-1 for M, which further improved the match.

Similarly to the null case, the estimated $E\chi^{2}$ was well approximated by our theoretical epxression under the independent SNP assumption as shown in Figure 4a, where the data points fall in the vicinity of the identity line. Panels b–d of the figure corroborate the linear relationship between the expected $\chi^{2}$ and 1/M, $h_{\delta }^{2}$, and N, respectively, as predicted by our equation (4).

### Observed inflation in TWAS with actual genotype data

To assess the practical relevance of this inflation in a traditional TWAS, we computed the association statistic between actual predicted expression levels and null polygenic target traits. We used genotype data from unrelated White British individuals in the UK Biobank with sample sizes of 1,000, 5,000 and 10,000. We predicted expression levels of 7,131 genes in whole blood using the GTEx v8 prediction models (Barbeira et al., 2021). To generate null polygenic target traits, we simulated Y with heritability ranging from 0.01 to 0.99 using the same UK Biobank genotype data. We sampled the effect sizes $\delta_{k}$ and independent error term ϵ from independent standard normal distributions. For each combination of gene, heritability value, and sample size, we generated 1,000 independent simulated traits. We regressed out the first five genetic principal components from the simulated trait to avoid capturing associations due to population structure, which we found to be sufficient to account for population structure in our simulations. Finally, we regressed the residuals of the simulated traits against predicted expression levels and estimated the expected $\chi_{\text{twas}}^{2}$ statistics, averaging the results across the 1000 simulations.

**Figure 5:**
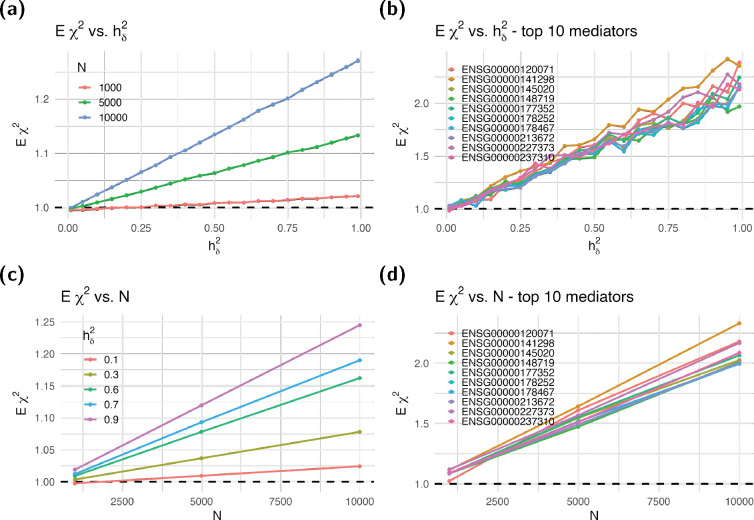
Expected $\chi^{2}$ in TWAS. We calculated the expected $\chi^{2}$ statistics in TWAS using simulated target traits with different heritability values $h_{\delta }^{2}$ of the target trait and sample sizes N. We averaged over 1000 simulated Y for a given gene, heritability, and sample size to estimate the ${E\chi }^{2}$. Panel (**a**) shows the expected $\chi^{2}$ averaged over genes as a function of the heritability $h_{\delta }^{2}$, with different lines representing different sample sizes N. Panel (**b**) shows the expected $\chi^{2}$ statistics for each gene against the heritability $h_{\delta }^{2}$ of the target trait averaged over different sample sizes. Colored lines in this panel represent selected genes; we highlight the 10 genes with the highest inflation. The lines in this panel are closely linear as predicted by the formula in equation (4). Panel (**c**) shows the expected $\chi^{2}$ against the sample size N, with different colors showing different heritability values $h_{\delta }^{2}$. Panel (**d**) shows the $\chi^{2}$ statistics for each gene against the sample size N averaged over different heritability values $h_{\delta }^{2}$. The lines in this panel are also closely linear, consistent with the formula in equation (4).

Figure 5 shows the resulting average $\chi_{\text{twas}}^{2}$, which show a linear dependence on the heritability of the target trait and the sample size of the association consistent with equation (4). Panels (a) and (c) show the average $\chi_{\text{twas}}^{2}$ for all genes, whereas panels (b) and (d) show the average $\chi_{\text{twas}}^{2}$ for the top 10 genes with the highest inflation. Similar results were obtained for other mediators such as metabolites and brain features (Figure S7 and S8), showing the robustness of equation (4) to mediators with differing genetic architecture.

Next, we used the average $\chi^{2}$ to estimate of the factor $\Phi_{\text{gene}}$ for 7,131 genes expression in whole blood. We regressed the average $\chi_{\text{twas}}^{2}$ on the product of sample size and heritability $\left(Y\sim N\;h_{\delta }^{2}\right)$. To borrow information across genes, we used a mixed effects model with a common intercept for all genes and random slopes for each gene. For a realistic scenario with biobank-level sample size of 500,000 individuals and heritability of 0.50, the noncentrality parameter $\left(Nh_{\delta }^{2}\Phi \right)$ would be 5, illustrated as an example in Figure 1. We also applied the same procedure to 1,156 plasma metabolites and 308 diffusion MRI brain features.

Reassuringly, the intercepts for each class of mediator traits were all very close to one: 0.995 (s.e. 10^−4^) for gene expression, 0.994 (s.e. 3 × 10^−4^) for metabolites, and 0.982 (s.e. 5 × 10^−4^) for brain features.

**Figure 6:**
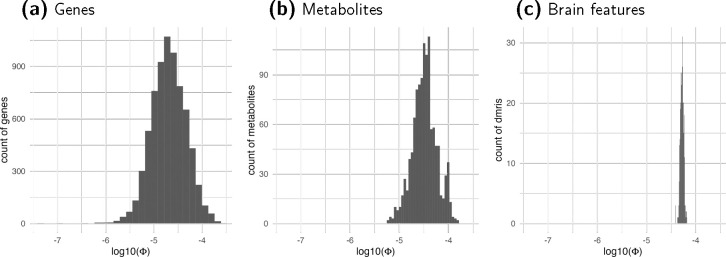
Distribution of Estimated Φ. Inflation factors Φ for gene expression, metabolites, and brain features (diffusion MRI) are shown in the log10 scale. The factor Φ for each mediator is estimated using the average $\chi^{2}$ statistics of the association between genetically predicted mediator and 1,000 simulated target traits for each combination of heritability of target trait $h_{\delta }^{2}$ and sample size N. The slope of the regression of $E\chi^{2}$ on $Nh_{\delta }^{2}$ is used to estimate Φ. Most values (78% of genes, 94% of metabolites, 100% of brain features) are in the range of 10^−5^.

The estimates of Φ, as indicated by the slope, varied across genes, metabolites, and brain features. The estimated Φ for gene expression had the largest variation (0 – 2 × 10^−4^, median of 2 × 10^−5^, followed by metabolites (0 – 1.5 × 10^−4^, median of 3.3 × 10^−5^), and, finally, brain features as shown in Figure 6. Both metabolites and brain features (3.9 × 10^−5^ – 6.5 × 10^−5^, median of 5.3 × 10^−5^) are more polygenic than gene expression. The majority (78% of genes, 94% of metabolites, 100% of brain features) of the mediating traits had Φ values in the 10^−5^) range. We obtained negative estimates of Φ for 34 genes, 3 metabolites, and 0 brain features; we set these negative estimates to 0, the lowest possible value of Φ.

### Proposed solution to the inflation problem

To account for the inflation in the PrediXcan software, the p-values will be calculated using the noncentral $\chi^{2}$ distribution with noncentrality parameter $Nh_{\delta }^{2}\Phi$. For example, in R the command would be <Monospace>pchisq(chi2, ncp=N*h2Y*phi, df=1, lower.tail=FALSE), </Monospace> where chi2 is the $\chi_{\text{twas}}^{2}\;statistic=Z^{2}$, ncp is the noncentrality parameter, df is the degrees of freedom, h2Y is the heritability of the target trait, and phi is the estimated noncentrality parameter for the gene (or the mediator more generally).

To help users of the PrediXcan framework implement the correction, we will make the corrected values the default output from the software, and we will share the estimated Φ in the same database as the prediction weights. When the user performs the the actual TWAS analysis with GWAS data, the software will estimate the heritability of the GWAS trait. The sample size will have to be provided. Then, the noncentrality parameter will be calculated using $1+h^{2}N{\;\Phi }_{\text{gene}}$, where $h^{2}$ is the heritability of the GWAS trait and N is the sample size of the GWAS.

## Discussion

We quantified the effect of polygenicity and prediction error of mediating traits in TWAS and related methods. To accomplish this, we derived a general closed-form asymptotic expression for the inflation, the validity of which was confirmed by simulations and TWAS associations based on real genetically predicted mediators of varying genetic architecture.

We found that the inflation is driven by the polygenicity of the target trait and not the polygenicity of the mediator trait. This is important to note because cis gene expression levels are mostly sparse ( Wheeler et al. (2016)). We also demonstrated that the inflation is not affected by the precision (or, equivalently, the uncertainty) of the prediction of the mediator trait under the null.

The closed-form solution for independent SNPs provided us with useful insights of the cause of the inflation: as the number of independent causal SNPs increases, the factor Φ trends to zero. However, the sample size increase offsets that trend. Polygenicity in the target trait is necessary for inflation to occur, since inflation is zero when the polygenic component of Y is zero.

We also show how to compute the correct p-values by estimating the parameters of the noncentral $\chi^{2}$ distribution. We will share the estimated parameters in the same database as the prediction weights and provide updated PrediXcan software to calculate the correct p-values.

The fact that the uncertainty in prediction does not increase type I error means that when we leave out mediators based on their prediction performance—a common practice in TWAS—we can be less stringent with the threshold. Adding more noisy predictors reduces the power due to multiple testing but will not contribute to inflation. The decision on which parameters to prioritize will depend on the goal of the study. For example, mediators with suspected functional roles but low heritability, and therefore predicted with larger uncertainty, need not be excluded from the TWAS analysis for fear of increasing the false positive rate. This could increase the discovery yield of TWAS and related methods without increasing the false positives.

In summary, this study demonstrates the following key conclusions: 1) Polygenicity of the target trait induces inflation in the test statistics regardless of the genetic architecture of the mediating trait. 2) Uncertainty in the prediction of the mediator does not cause inflation. 3) Uncertainty in the prediction of the mediator reduces the power of the test. 4) LD is not necessary for the inflation to occur (our simulations were done using independent SNPs). 5) The inflation can be corrected by using the noncentral $\chi^{2}$ distribution with noncentrality parameter $N\;h_{\delta }^{2}\;\Phi$, where the factor Φ can be pre-calculated independent of the GWAS.

We have attempted to use realistic assumptions and real genotype data and prediction models for mediators to illustrate the validity of our derivations. However, our study has several limitations: We made several assumptions to streamline the theoretical derivation. These are commonly used assumptions, but they may not hold in practice (e.g., the independence of the effect sizes and the noise terms). However, the results based on actual genotype data and prediction models for gene expression, metabolites, and brain features suggest that prediction results are likely to be robust to these assumptions. We assume an additive infinitesimal model for the target trait to simplify the theoretical derivation, but traits may deviate from this model in practice. Improvements on the estimation of the inflation factor Φ could be made for different genetic architectures. Even so, we expect that the infinitesimal model will be conservative. Our model does not include gene–gene and gene–environment interactions. Their effect on inflation is not clear and will need to be addressed in future work. Finally, our derivations used a linear regression framework, but many GWAS are performed using logistic regression. Linear regression results are a good approximation for logistic regression when the case control ratio is balanced. Hence, we expect our results to be broadly applicable for balanced designs but will need adjustments for unbalanced designs.

## Methods

### Derivation of the distribution of the $\chi^{2}$ statistic under the null

Here we derive the formula (4) restated below for completeness.

$$E\chi_{\text{twas}\;}^{2}\approx 1+Nh_{\delta }^{2}\frac{\Phi }{1\;-\;\beta^{2}\tau^{2}}+N\frac{\beta^{2}}{1\;-\;\beta^{2}\tau^{2}}\tau^{2}$$

We also restate the definitions of the variables used in the formula. To simplify notation and derivation, we use the vector form of the model.

Y=βT+X⋅δ+ϵ

T=X⋅γ

$$\tilde{T}=X\;\cdot \;(\gamma +e)=X\;\cdot \;\tilde{\gamma }$$

where δ,γ, $e_{\gamma }$, and ϵ all normally distributed and independent of each. $\tilde{\gamma }$ is defined as $\gamma +e_{\gamma }.\;\gamma ,\;\delta$ are M-dimensional vectors with elements $\gamma_{k},\;\delta_{k},\;X$ is N×M matrix, ϵ is N-dimensional vector, and $e_{\gamma }$ is M-dimensional vector. Let M be the number of causal SNPs for target trait Y,N be the sample size, and $h_{\beta }^{2}=\beta^{2}$ be the heritability of the target trait explained by the mediator T.

We define the sample (M×M) LD matrix as

$$R\;:=\frac{X^{\prime }\;\cdot \;X}{N}$$

and its large N limit Σ.

Without loss of generality (WLOG), we assume Y,T, and $X_{k}$ have mean = 0 and variance = 1. For simplicity we ignore the difference between N and N-1, since we are interested in the case where the sample size N is large.

(10) $$1=var(Y)\approx \hat{var}(Y)\;=Y^{\prime }\;\cdot \;Y/N$$

(11) $$1=var(T)\approx \hat{var}(T)=T^{\prime }\;\cdot \;T/N$$

(12) $$1=var\left(X_{k}\right)\approx \hat{var}\left(X_{k}\right)=X_{k}^{\prime }\;\cdot \;X_{k}/N$$

We list here additional assumptions and facts used in the derivation.

- X, δ, γ, ϵ, $e_{\gamma }$ are all independent of each other.
- δ, ϵ, γ, $e_{\gamma }$ are all normally distributed, although we can relax this assumption and only need to require that they are well-behaved so that central limit theorems apply. $\gamma_{k}$ ’s can have a positive probability of being 0 to allow sparse architecture for the mediator.
- M,N≫1, hence N-1≈N, M-1≈M.
- We assume that we know X, γ, $\tilde{\gamma }$, which means that we are conditioning on these variables unless stated otherwise. This also implies that we are conditioning on T and $\tilde{T}$.
- We considered the case where the sample size is larger than the number of independent causal SNPs for Y, i.e., N>M. With GWAS studies increasing in sample size, this is the most likely scenario.

We defined $\tau^{2}$ as the ratio of the variances of T and $\tilde{T}$, but we related it to the sample variances $\tau^{2}\approx \hat{var}(T)/\hat{var}(\tilde{T})=1/\hat{var}(\tilde{T})$, therefore

(13) $$\hat{var}(\tilde{T})\approx \frac{1}{\tau^{2}}$$

Furthermore,

(14) $$\begin{array}{l} \;\frac{1}{\tau^{2}}\approx {\tilde{\gamma }}^{\prime }\;\cdot \;R\;\cdot \;\tilde{\gamma }\\ \text{since,}\;\;\frac{1}{\tau^{2}}\approx \hat{var}(\tilde{T})\\ \;={\tilde{T}}^{\prime }\;\cdot \;\tilde{T}/N\\ \;={\tilde{\gamma }}^{\prime }\;\cdot \;X^{\prime }\;\cdot \;X\;\cdot \;\tilde{\gamma }/N\\ \;={\tilde{\gamma }}^{\prime }\;\cdot \;R\;\cdot \;\tilde{\gamma } \end{array}$$

**The estimated** $\overset{ˆ}{\beta }$ **in a TWAS is** (Weisberg, 2005)

(15) $$\overset{ˆ}{\beta }\;={\left({\tilde{T}}^{\prime }\;\cdot \;\tilde{T}\right)}^{-1}{\tilde{T}}^{\prime }\;\cdot \;Y\;\;=\frac{\tau^{2}}{N}\left[{\tilde{T}}^{\prime }\;\cdot \;(T\beta +X\;\cdot \;\delta +\epsilon )\right]\;\;∵{\tilde{T}}^{\prime }\;\cdot \;\tilde{T}=N\;\hat{var}(\tilde{T})=N/\tau^{2}\;\text{see}\;\text{eq}\;(13)$$

(16) $$=\frac{\tau^{2}}{N}\left[{\tilde{T}}^{\prime }\;\cdot \;T\beta +{\tilde{T}}^{\prime }\;\cdot \;X\;\cdot \;\delta +{\tilde{T}}^{\prime }\;\cdot \;\epsilon \right]$$

(17) $$\approx \frac{\tau^{2}}{N}\left[N\;\beta +{\tilde{T}}^{\prime }\;\cdot \;X\;\cdot \;\delta +{\tilde{T}}^{\prime }\;\cdot \;\epsilon \right]\;\;∵{\tilde{T}}^{\prime }\;\cdot \;T\approx N\;see\;eq\;(19)$$

(18) $$\overset{ˆ}{\beta }\approx \frac{\tau^{2}}{N}\left[N\;\beta +{\tilde{T}}^{\prime }\;\cdot \;X\;\cdot \;\delta +{\tilde{T}}^{\prime }\;\cdot \;\epsilon \right]$$

Using

(19) $${\tilde{T}}^{\prime }\;\cdot \;T={\left(T+E_{T}\right)}^{\prime }\;\cdot \;T=T^{\prime }\;\cdot \;T+T^{\prime }\;\cdot \;E_{T}=N+O_{p}(\sqrt{N})\approx N$$

**The expected value of** $\overset{ˆ}{\beta }$ **is**

(20) $$E\;\overset{ˆ}{\beta }=\tau^{2}\;\beta$$

since the expected value of the last two terms in (18) equal to zero, i.e. $E\;\left[{\tilde{T}}^{\prime }\;\cdot \;X\;\cdot \;\delta +{\tilde{T}}^{\prime }\;\cdot \;\epsilon \right]={\tilde{T}}^{\prime }\;\cdot \;X\;\cdot \;E\;\delta +{\tilde{T}}^{\prime }\;\cdot \;E\;\epsilon =0$.

**Next, we show that** $\overset{ˆ}{\beta }$ **tends to** $\tau^{2}\beta$ **for large** N.

(21) $$\overset{ˆ}{\beta }⟶\tau^{2}\beta \;\;\text{for}\;N\gg 1$$

since in equation (18) $X\cdot \delta /N=O_{p}(1/\sqrt{N})$ and ${\tilde{T}}^{\prime }\cdot \epsilon /N=O_{p}(1/\sqrt{N})$ for large N using $X⫫\delta \tilde{T}⫫\epsilon$.

We want to calculate the $\chi_{\text{twas}}^{2}$ statistic. For that we need the TWAS-estimated variance of $\overset{ˆ}{\beta }$. From standard regression results (Weisberg, 2005), the variance of $\overset{ˆ}{\beta }$ is estimated as

$$\hat{var}(\overset{ˆ}{\beta })=\frac{\hat{var}\left(\epsilon_{\text{twas}}\right)}{{\tilde{T}}^{\prime }\tilde{T}}$$

where the estimate of the variance of the error term $\hat{var}\left(\epsilon_{\text{twas}}\right)$ is calculated as the residual some of squares (RSS) divided by N-1 (Weisberg, 2005).

$$RSS=(Y-\tilde{T}\;\overset{ˆ}{\beta }{)}^{\prime }(Y-\tilde{T}\;\overset{ˆ}{\beta })\;\;=Y^{\prime }Y-2{\overset{ˆ}{\beta }}^{\prime }{\tilde{T}}^{\prime }Y+{\overset{ˆ}{\beta }}^{\prime }{\tilde{T}}^{\prime }\tilde{T}\overset{ˆ}{\beta }\;\;=Y^{\prime }Y-{\overset{ˆ}{\beta }}^{2}{\tilde{T}}^{\prime }\tilde{T}\;\;∵(\overset{ˆ}{\beta }{\tilde{T}}^{\prime }\tilde{T})={\tilde{T}}^{\prime }Y\;\;=N-N{\overset{ˆ}{\beta }}^{2}/\tau^{2}\;\;\approx N-N\beta^{2}\tau^{4}/\tau^{2}\;∵{\overset{ˆ}{\beta }}^{2}\approx {\left(\beta \tau^{2}\right)}^{2}∵(21)\;\;=N\left(1-\beta^{2}\tau^{2}\right)$$

(22) $$\hat{var}\left(\epsilon_{\text{twas}}\right)=\frac{RSS}{N\;-\;1}\approx \frac{N\left(1\;-\;\beta^{2}\tau^{2}\right)}{N\;-\;1}\approx 1-\beta^{2}\tau^{2}$$

The variance of $\overset{ˆ}{\beta }$ used in TWAS, which is unaware of the polygenic term X⋅δ, is

$${v\hat{a}r}_{\text{twas}\;}(\overset{ˆ}{\beta })=\frac{v\hat{a}r\left(\epsilon_{\text{twas}\;}\right)}{{\tilde{T}}^{\prime }\tilde{T}}=\left(1-\beta^{2}\tau^{2}\right)\frac{\tau^{2}}{N}\;∵(15)$$

This variance, ${v\hat{a}r}_{\text{twas}}(\overset{ˆ}{\beta })$, is not the actual variance of $\overset{ˆ}{\beta }$ when the target trait is polygenic, which is the source of the inflation.

Hence, $\chi_{\text{twas}}^{2}$ is given by

$$\chi_{\text{twas}\;}^{2}=\frac{{\overset{ˆ}{\beta }}^{2}}{{\hat{var}}_{\text{twas}}(\overset{ˆ}{\beta })}\approx {\overset{ˆ}{\beta }}^{2}\frac{1}{1\;-\;\beta^{2}\tau^{2}}\frac{N}{\tau^{2}}$$

The $\chi^{2}$ statistics in TWAS is given by

$$\chi_{\text{twas}\;}^{2}\;={\overset{ˆ}{\beta }}^{2}\frac{1}{1\;-\;\beta^{2}\tau^{2}}\frac{N}{\tau^{2}}\;\;={\left(\frac{\tau^{2}}{N}\right)}^{2}{\left[N\;\beta +{\tilde{T}}^{\prime }\;\cdot \;X\;\cdot \;\delta +{\tilde{T}}^{\prime }\;\cdot \;\epsilon \right]}^{2}\frac{1}{1\;-\;\beta^{2}\tau^{2}}\frac{N}{\tau^{2}}\;\;={\left[N\;\beta +{\tilde{T}}^{\prime }\;\cdot \;X\;\cdot \;\delta +{\tilde{T}}^{\prime }\;\cdot \;\epsilon \right]}^{2}\frac{1}{1\;-\;\beta^{2}\tau^{2}}\frac{\tau^{2}}{N}$$

Now, we take expectation of the $\chi^{2}$ stat to get the mean of the distribution.

$$E\;\chi_{\text{twas}\;}^{2}=E\;{\left[N\;\beta +{\tilde{T}}^{\prime }\;\cdot \;X\;\cdot \;\delta +{\tilde{T}}^{\prime }\;\cdot \;\epsilon \right]}^{2}\frac{1}{1\;-\;\beta^{2}\tau^{2}}\frac{\tau^{2}}{N}$$

Rearranging terms, we get

(23) $$\frac{N}{\tau^{2}}\left(1-\beta^{2}\tau^{2}\right)\;E\;\chi_{\text{twas}}^{2}=E\;{\left[N\;\beta +{\tilde{T}}^{\prime }\;\cdot \;X\;\cdot \;\delta +{\tilde{T}}^{\prime }\;\cdot \;\epsilon \right]}^{2}$$

When we expand the square of the terms between the brackets, all the cross terms have expectation 0 as shown below.

$$E\;[N\;\beta {\;\tilde{T}}^{\prime }\;\cdot \;X\;\cdot \;\delta ]=N\;\beta {\;\tilde{T}}^{\prime }\;\cdot \;X\;\cdot \;E\;[\delta ]=0\;∵E\;\delta =0\;E\;[N\;\beta {\tilde{\;T}}^{\prime }\;\cdot \;X\;\cdot \;\epsilon ]=N\;\beta \;{\tilde{T}}^{\prime }\;\cdot \;X\;\cdot \;E\;[\epsilon ]=0\;∵E\;\epsilon =0\;E\;[{\tilde{T}}^{\prime }\;\cdot \;X\;\cdot \;\delta \;\cdot \;{\tilde{T}}^{\prime }\;\cdot \;\epsilon ]=E\;E\;[{\tilde{T}}^{\prime }\;\cdot \;X\;\cdot \;\delta \;\cdot \;{\tilde{T}}^{\prime }\;\cdot \;\epsilon |\delta ]=E\;[{\tilde{T}}^{\prime }\;\cdot \;X\;\cdot \;\delta \;\cdot \;{\tilde{T}}^{\prime }\;\cdot \;E[\epsilon |\delta ]]=0\;\;∵E\left(\epsilon |\delta \right)=E\left(\epsilon \right)=0\;\;∵\epsilon ⫫\delta$$

So the expectation is given by the expected value of the squared terms

$$\left(23\right)=E(N\;\beta {)}^{2}+E{\;({\tilde{T}}^{\prime }\;\cdot \;X\;\cdot \;\delta )}^{2}+E{\left({\tilde{T}}^{\prime }\;\cdot \;\epsilon \right)}^{2}\;\;=N^{2}\beta^{2}+E\;({\tilde{T}}^{\prime }\;\cdot \;X\;\cdot \;\delta \;\cdot \;\delta^{\prime }\;\cdot \;X^{\prime }\;\cdot \;\tilde{T})+E({\tilde{T}}^{\prime }\;\cdot \;\epsilon \;\cdot \;\epsilon^{\prime }\;\cdot \;{\tilde{T}}^{\prime })\;\;∵({\tilde{T}}^{\prime }\;\cdot \;X\;\cdot \;\delta )={\left({\tilde{T}}^{\prime }\;\cdot \;X\;\cdot \;\delta \right)}^{\prime }\;\;∵({\tilde{T}}^{\prime }\;\cdot \;\epsilon )={\left({\tilde{T}}^{\prime }\;\cdot \;\epsilon \right)}^{\prime }\;=N^{2}\beta^{2}+{\tilde{T}}^{\prime }\;\cdot \;X\;\cdot \;E\;(\delta \;\cdot \;\delta^{\prime })\;\cdot \;X^{\prime }\;\cdot \;\tilde{T})+{\tilde{T}}^{\prime }\;\cdot \;E\;\left(\epsilon \;\cdot \;\epsilon^{\prime }\right)\;\cdot \;{\tilde{T}}^{\prime }\;\;=N^{2}\beta^{2}+{\tilde{T}}^{\prime }\;\cdot \;X\;\cdot \;\sigma_{\delta }^{2}I_{M}\;\cdot \;X^{\prime }\;\cdot \;\tilde{T}+{\tilde{T}}^{\prime }\;\cdot \;\sigma_{\epsilon }^{2}I_{N}\;\cdot \;\tilde{T}\;\;=N^{2}\beta^{2}+\sigma_{\delta }^{2}{\tilde{\gamma }}^{\prime }\;\cdot \;X^{\prime }\;\cdot \;X\;\cdot \;X^{\prime }\;\cdot \;X\;\cdot \;\tilde{\gamma }+\sigma_{\epsilon }^{2}{\tilde{T}}^{\prime }\;\cdot \;\tilde{T}\;\;=N^{2}\beta^{2}+N^{2}\frac{h_{\delta }^{2}}{M}{\tilde{\gamma }}^{\prime }\;\cdot \;R\;\cdot \;R\;\cdot \;\tilde{\gamma }+\sigma_{\epsilon }^{2}{\tilde{T}}^{\prime }\;\cdot \;\tilde{T}\;∵\;assume\;an\;infinitesimal\;model\;for\;\delta ,{i.e.\sigma }_{\delta }^{2}=\frac{h_{\delta }^{2}}{M}\;\;\;$$

(24) $$=N^{2}\beta^{2}+N^{2}\frac{h_{\delta }^{2}}{M}{\tilde{\gamma }}^{\prime }\;\cdot \;R^{2}\;\cdot \;\tilde{\gamma }+\sigma_{\epsilon }^{2}\frac{N}{\tau^{2}}\;\;∵{\tilde{T}}^{\prime }\;\cdot \;\tilde{T}=N/\tau^{2},$$

see eq(15)

Assuming a polygenic mediator,

$$\begin{array}{l} \left(24)\;\approx N^{2}\beta^{2}+N^{2}\frac{h_{\delta }^{2}}{M}tr\left(R^{2}\right)var(\tilde{\gamma })+\sigma_{\epsilon }^{2}\frac{N}{\tau^{2}}∵{\tilde{\gamma }}^{\prime }\;\cdot \;R^{2}\;\cdot \;\tilde{\gamma }\approx tr\left(R^{2}\right)var(\tilde{\gamma }),\;\text{see}\;(34\right)\\ \;\approx N^{2}\beta^{2}+\frac{N^{2}}{\tau^{2}}\frac{h_{\delta }^{2}}{M^{2}}tr\left(R^{2}\right)+\sigma_{\epsilon }^{2}\frac{N}{\tau^{2}}\;∵var(\tilde{\gamma })\approx \frac{1}{M\tau^{2}},\;\text{see}\;(36) \end{array}$$

Multiplying both sides by $\tau^{2}/N$

$$\left(1-\beta^{2}\tau^{2}\right)\;E\;\chi_{twas}^{2}\;=\frac{\tau^{2}}{N}\left[N^{2}\beta^{2}+\frac{N^{2}}{\tau^{2}}\frac{h_{\delta }^{2}}{M^{2}}tr\left(R^{2}\right)+\sigma_{\epsilon }^{2}\frac{N}{\tau^{2}}\right]\;\;=N\beta^{2}\tau^{2}+Nh_{\delta }^{2}\frac{tr\left(R^{2}\right)}{M^{2}}+\sigma_{\epsilon }^{2}$$

Using $\sigma_{\epsilon }^{2}=1-\beta^{2}\tau^{2}-h_{\delta }^{2}-\beta^{2}\left(1-\tau^{2}\right)$, see (??)

$$\left(1-\beta^{2}\tau^{2}\right)\;E\chi_{\text{twas}\;}^{2}\;=N\beta^{2}\tau^{2}+N\frac{h_{\delta }^{2}}{M^{2}}tr\left(R^{2}\right)+\left(1-\beta^{2}\tau^{2}\right)-h_{\delta }^{2}-\beta^{2}\left(1-\tau^{2}\right)\;\;=\left(1-\beta^{2}\tau^{2}\right)+N\beta^{2}\tau^{2}-\beta^{2}\left(1-\tau^{2}\right)+N\;h_{\delta }^{2}\left(\frac{tr\left(R^{2}\right)}{M^{2}}-\frac{1}{N}\right)\;\;=\left(1-\beta^{2}\tau^{2}\right)+N\beta^{2}\tau^{2}\left(1-\frac{1\;-\;\tau^{2}}{N\tau^{2}}\right)+N\;h_{\delta }^{2}\left(\frac{tr\left(R^{2}\right)}{M^{2}}-\frac{1}{N}\right)\;\;\approx \left(1-\beta^{2}\tau^{2}\right)+N\beta^{2}\tau^{2}+N\;h_{\delta }^{2}\left(\frac{tr\left(R^{2}\right)}{M^{2}}-\frac{1}{N}\right)\;\;∵\tau^{2}>0∴1-\frac{1\;-\;\tau^{2}}{N\tau^{2}}\approx 1\;\text{when}\;N\gg 1$$

Note τ>0 so for large $N,1-\frac{1-\tau^{2}}{N\tau^{2}}\approx 1$

$$E\chi_{\text{twas}\;}^{2}\approx 1+\frac{Nh_{\delta }^{2}}{1\;-\;\beta^{2}\tau^{2}}\Phi_{\text{polygenic}}+\frac{N\beta^{2}\tau^{2}}{1\;-\;\beta^{2}\tau^{2}}$$

where $\Phi_{\text{polygenic}}$ is defined as

(25) $$\Phi_{\text{polygenic}\;}:=\frac{tr\left(R^{2}\right)}{M^{2}}-\frac{1}{N}\;\;\text{polygenic}\;\text{mediator}$$

### Derivation of Φ and $E\chi^{2}$ when SNPs are independent

We calculate $tr\left(R^{2}\right)$ using the asymptotic distribution of the eigenvalues of $R^{2}$. When SNPs are independent and M and N are large but comparable, i.e. M/N converges to a number that is finite and greater than 0, the eigenvalues of R follow a Marchenko-Pastur distribution (Bai and Silverstein, 2010). The trace of $R^{2}$ can be approximated by the second moment of the Marchenko-Pastur distribution multiplied by M (see 28). We use Lemma 3.1 in (Bai and Silverstein, 2010), which states that the kth moment is

(26) $$\text{E}\lambda^{k}=\sum_{r}^{k-1}\frac{1}{r\;+\;1}\left(\begin{array}{c} k\\ r \end{array}\right)\left(\begin{array}{c} k-1\\ r \end{array}\right){\left(\frac{M}{N}\right)}^{r}$$

In the case of k=2, eq.(26) becomes

$$1+\frac{M}{N}$$

so that

$$tr\left(R^{2}\right)=M+\frac{M^{2}}{N}$$

$$\Phi \;=\frac{1}{M}\;\cdot \;\frac{tr\left(R^{2}\right)}{M}-\frac{1}{N}\;\;\approx \frac{1}{M}(1+\frac{M}{N})-\frac{1}{N}\;\;=\frac{1}{N}+\frac{1}{M}-\frac{1}{N}\;\;=\frac{1}{M}$$

Therefore, (4) becomes to the following when SNPs are independent.

Since these derivations are based on asymptotic approximations with N≥1 and M≫1, they do not distinguish between M or M-1.

(27) $$E\chi_{\text{twas}}^{2}\approx 1+Nh_{\delta }^{2}\;\cdot \;\left[\frac{1}{M}\right]\;\;\text{for}\;\text{indep}\;\text{SNPs}\;\text{under}\;\text{the}\;\text{null}$$

### Derivation of useful results

#### Calculating $\gamma^{\prime }\;\cdot \;R\;\cdot \;\gamma$ and $\gamma^{\prime }\;\cdot \;R^{2}\;\cdot \;\gamma$ under fully polygenic T

By “fully polygenic T”, we mean that $\gamma_{i}\sim_{\text{iid}}N\left(0,\sigma_{\gamma }^{2}\right)$ for each variant i across the genome. Here we want to show that $\gamma^{\prime }A\gamma =\sigma_{\gamma }^{2}tr(A)$ for =R, $R^{2}$. We start with A=R.

R is symmetric so it can be eigenvalue-decomposed

$$R=C\;\cdot \;\Lambda \;\cdot \;C^{\prime }$$

where C is the matrix of eigenvectors of R as columns and is orthonormal $C^{\prime }C=I.\;\Lambda$ is a diagonal matrix with the eigenvalues of R, $\lambda_{k}$.

$$\gamma^{\prime }\;\cdot \;R\;\cdot \;\gamma \;=\gamma^{\prime }C\;\cdot \;\Lambda \;\cdot \;C^{\prime }\gamma \;\;=\gamma_{C}^{\prime }\;\cdot \;\Lambda \;\cdot \;\gamma_{C}\;\;\text{with}\;\gamma_{C}\;:=C^{\prime }\;\cdot \;\gamma \;\;=\sum_{k}\;\gamma_{C,k}^{2}\lambda_{k}$$

Since the eigenvalues of $R^{2}$ are the squares of the eigenvalues of R, we have

$$\gamma^{\prime }\;\cdot \;R^{2}\;\cdot \;\gamma \;=\gamma^{\prime }C\Lambda^{2}C^{\prime }\gamma \;\;=\gamma_{C}^{\prime }\Lambda^{2}\gamma_{C}\;\;=\sum_{k}\;\gamma_{C,k}^{2}\lambda_{k}^{2}$$

Furthermore

(28) $$\sum_{k}\gamma_{C,k}^{2}\lambda_{k}^{2}\approx M\;\text{E}\;\gamma_{C,k}^{2}\lambda_{k}^{2}\;∵\;\text{law}\;\text{large}\;\text{numbers}\;\;\text{E}\;\gamma_{C,k}^{2}\text{E}\lambda_{k}^{2}\;∵\gamma_{C}\;\text{and}\;\lambda \;\text{are}\;\text{indep}\;\;\text{=}\sigma_{\gamma }^{2}\;M\;\text{E}\;\lambda_{k}^{2}\;\;\text{since}\;\text{var}\left(\gamma_{C}\right)=\text{var}\left(C^{\prime }\gamma \right)=\sigma_{\gamma }^{2}∵C\;\text{orthonormal}\;\;\approx \sigma_{\gamma }^{2}\text{tr}\left(R^{2}\right)$$

(29) $$\gamma^{\prime }\;\cdot \;R^{2}\;\cdot \;\gamma \approx \sigma_{\gamma }^{2}tr\left(R^{2}\right)\;\;\text{polygenic}\;\text{mediator}$$

Similarly, we get

$$\begin{array}{l} \gamma^{\prime }\;\cdot \;R\;\cdot \;\gamma \;\approx M\sigma_{\gamma }^{2}E\lambda_{k}\\ \;=\sigma_{\gamma }^{2}ME\lambda_{k}\\ \;\approx \sigma_{\gamma }^{2}tr(R)\\ \;=\sigma_{\gamma }^{2}M \end{array}$$

(30) $$\gamma^{\prime }\;\cdot \;R\;\cdot \;\gamma \approx \sigma_{\gamma }^{2}M\;polygenic\;mediator$$

**Proof that** $\gamma^{\prime }\;\cdot \;R\;\cdot \;\gamma =1$

$$\begin{array}{l} 1\;=\hat{var}(T)\\ \;=\hat{var}(X\;\cdot \;\gamma )\\ \;=\gamma^{\prime }\;\cdot \;X^{\prime }\;\cdot \;X\;\cdot \;\gamma /N\\ \;=\gamma^{\prime }\;\cdot \;R\;\cdot \;\gamma \end{array}$$

so we have

(31) $$\gamma^{\prime }\;\cdot \;R\;\cdot \;\gamma =1$$

**Proof that** $\sigma_{\gamma }^{2}=1/M$ From eq (30) and (31), we have $\sigma_{\gamma }^{2}M\approx \gamma^{\prime }\;\cdot \;R\;\cdot \;\gamma =1$

(32) $$\sigma_{\gamma }^{2}=1/M$$

Proof that tr(R)=M

(33) $$\text{tr}\left(R\right)=\sum_{k}R_{kk}=\sum_{k}\sum_{i}X_{ki}^{\prime }X_{ik}/N=\sum_{k}\sum_{i}X_{ik}^{2}/N=\sum_{k}\hat{\text{var}}\left(X_{k}\right)=\sum_{k}1=M$$

Analogous derivation for $\tilde{\gamma }$ yields

(34) $${\tilde{\gamma }}^{\prime }\;\cdot \;R^{2}\;\cdot \;\tilde{\gamma }=var(\tilde{\gamma })tr\left(R^{2}\right)$$

(35) $${\tilde{\gamma }}^{\prime }\;\cdot \;R\;\cdot \;\tilde{\gamma }=var(\tilde{\gamma })tr(R)$$

**Proof that** $\mathrm{var}(\tilde{\gamma })=1/\left(\tau^{2}M\right)$

$$\begin{array}{l} \frac{1}{\tau^{2}}=\hat{var}(\tilde{T})\;\text{see}\;\text{(13)}\;\\ \;={\tilde{T}}^{\prime }\;\cdot \;\tilde{T}\\ \;={\tilde{\gamma }}^{\prime }\;\cdot \;X^{\prime }\;\cdot \;X\;\cdot \;\tilde{\gamma }/N\\ \;={\tilde{\gamma }}^{\prime }\;\cdot \;R\;\cdot \;\tilde{\gamma }\\ \;=\mathrm{var}\left(\tilde{\gamma }\right)\mathrm{tr}\left(R\right)\;∵(35)\\ \;=\mathrm{var}\left(\tilde{\gamma }\right)M\;∵(33) \end{array}$$

therefore

(36) $$var(\tilde{\gamma })=1/\left(\tau^{2}M\right)$$

### Generalization of inflation formula to sparse mediators

Next, we show that the formula for the TWAS $\chi^{2}$ statistics in equation (24) holds for both polygenic and sparse mediators. However, when the mediator is a sparse mediator, the approximate identities - such as $\tilde{\gamma }\;\cdot \;R^{2}\;\cdot \;\tilde{\gamma }\approx tr\left(R^{2}\right)var(\tilde{\gamma })$ and $var(\tilde{\gamma })\approx \frac{1}{M\gamma^{2}}$ - will not work since they rely on the fact that there are a large number of variants to sum over and M representing the number of variants in the mediator is equal to the number of variants in the polygenic Y.

Instead, to include a sparse mediator into the scope, we can keep $\tilde{\gamma }$ as is in the derivation and such result will apply to both polygenic and sparse mediators. Recall that the equation 24 is as follow and $\tilde{\gamma }$ can take any value, i.e. for a sparse mediator, most entries in $\tilde{\gamma }$ equals to zero.

(37) $$(24)=N^{2}\beta^{2}+N^{2}h_{\delta }^{2}\frac{{\tilde{\gamma }}^{\prime }\;\cdot \;R^{2}\;\cdot \;\tilde{\gamma }}{M}+\sigma_{\epsilon }^{2}\frac{N}{\tau^{2}}$$

Similar to the derivation for the polygenic case,

$$\left(1-\beta^{2}\tau^{2}\right)E\chi_{\text{twas}\;}^{2}\;=\frac{\tau^{2}}{N}\left[N^{2}\beta^{2}+N^{2}\frac{h_{\delta }^{2}}{M}{\tilde{\gamma }}^{\prime }\;\cdot \;R^{2}\;\cdot \;\tilde{\gamma }+\sigma_{\epsilon }^{2}\frac{N}{\tau^{2}}\right]\;\;=N\beta^{2}\tau^{2}+N\tau^{2}\frac{h_{\delta }^{2}}{M}\;\cdot \;{\tilde{\gamma }}^{\prime }\;\cdot \;R^{2}\;\cdot \;\tilde{\gamma }+\sigma_{\epsilon }^{2}\;\;\approx N\beta^{2}\tau^{2}+N\frac{h_{\delta }^{2}}{M}\;\cdot \;\frac{{\tilde{\gamma }}^{\prime }\;\cdot \;R^{2}\;\cdot \;\tilde{\gamma }}{{\tilde{\gamma }}^{\prime }\;\cdot \;R\;\cdot \;\tilde{\gamma }}+\sigma_{\epsilon }^{2},\;\text{see}\;(14)\;\;=N\beta^{2}\tau^{2}+N\frac{h_{\delta }^{2}}{M}\;\cdot \;\frac{{\tilde{\gamma }}^{\prime }\;\cdot \;R^{2}\;\cdot \;\tilde{\gamma }}{{\tilde{\gamma }}^{\prime }\;\cdot \;R\;\cdot \;\tilde{\gamma }}+\left(1-\beta^{2}\tau^{2}\right)-h_{\delta }^{2}-\beta^{2}\left(1-\tau^{2}\right)\;\;=\left(1-\beta^{2}\tau^{2}\right)+N\beta^{2}\tau^{2}-\beta^{2}\left(1-\tau^{2}\right)+Nh_{\delta }^{2}\left(\frac{1}{M}\;\cdot \;\frac{{\tilde{\gamma }}^{\prime }\;\cdot \;R^{2}\;\cdot \;\tilde{\gamma }}{{\tilde{\gamma }}^{\prime }\;\cdot \;R\;\cdot \;\tilde{\gamma }}-\frac{1}{N}\right)\;\;=\left(1-\beta^{2}\tau^{2}\right)+N\beta^{2}\tau^{2}\left(1-\frac{1\;-\;\tau^{2}}{N\tau^{2}}\right)+Nh_{\delta }^{2}\left(\frac{1}{M}\;\cdot \;\frac{{\tilde{\gamma }}^{\prime }\;\cdot \;R^{2}\;\cdot \;\tilde{\gamma }}{{\tilde{\gamma }}^{\prime }\;\cdot \;R\;\cdot \;\tilde{\gamma }}-\frac{1}{N}\right)\;\;\approx \left(1-\beta^{2}\tau^{2}\right)+N\beta^{2}\tau^{2}+Nh_{\delta }^{2}\left(\frac{1}{M}\;\cdot \;\frac{{\tilde{\gamma }}^{\prime }\;\cdot \;R^{2}\;\cdot \;\tilde{\gamma }}{{\tilde{\gamma }}^{\prime }\;\cdot \;R\;\cdot \;\tilde{\gamma }}-\frac{1}{N}\right)$$

The last approximation, $(1-\frac{1-\tau^{2}}{N\tau^{2}})\approx 1$, is obtained as suggested by the derivation of the polygenic case.

So, for **any** mediators, our main result still holds

$$E\chi_{\text{twas}\;}^{2}\approx 1+\frac{Nh_{\delta }^{2}}{1\;-\;\beta^{2}\tau^{2}}\Phi_{R}+\frac{N\beta^{2}\tau^{2}}{1\;-\;\beta^{2}\tau^{2}}\;\;\text{sparse}\;\text{and}\;\text{polygenic}\;\text{mediator}\;$$

whereas to accommodate sparse T, we define $\Phi_{R}$ as

(38) $$\Phi_{R}\;:=\frac{1}{M}\frac{{\tilde{\gamma }}^{\prime }\;\cdot \;R^{2}\;\cdot \;\tilde{\gamma }}{{\tilde{\gamma }}^{\prime }\;\cdot \;R\;\cdot \;\tilde{\gamma }}-\frac{1}{N}$$

### Proof that $\Phi_{R}\approx \frac{1}{M}\frac{{\tilde{\gamma }}^{\prime }\cdot \Sigma^{2}\cdot \tilde{\gamma }}{{\tilde{\gamma }}^{\prime }\cdot \Sigma \cdot \tilde{\gamma }}\;and\;using\;this\;for\;the\;final\;version\;of\;\Phi$

We can further simplify the expression of Φ by noting that the numerator and denominator of

$$\frac{{\tilde{\gamma }}^{\prime }\;\cdot \;R^{2}\;\cdot \;\tilde{\gamma }}{{\tilde{\gamma }}^{\prime }\;\cdot \;R\;\cdot \;\tilde{\gamma }}$$

converge to their expectations as N increases. The sample covariance R follows a Wishart distribution: $N\;\cdot \;R\sim W_{M}(\Sigma ,\;N)$. Therefore ${\tilde{\gamma }}^{\prime }\;\cdot \;R^{2}\;\cdot \;\tilde{\gamma }$ and ${\tilde{\gamma }}^{\prime }\;\cdot \;R\;\cdot \;\tilde{\gamma }$ converge in distribution to their means as N goes to infinity. So we can approximate the ratio with the ratio of the expectations, using the corollary of the Slutsky’s theorem (Ferguson, 1996). We use $E_{R}$ to indicate that R is being integrated out.

(39) $$\begin{array}{l} \frac{{\tilde{\gamma }}^{\prime }\;\cdot \;R^{2}\;\cdot \;\tilde{\gamma }}{{\tilde{\gamma }}^{\prime }\;\cdot \;R\;\cdot \;\tilde{\gamma }}\;\approx \frac{E_{R}\left[{\tilde{\gamma }}^{\prime }\;\cdot \;R^{2}\;\cdot \;\tilde{\gamma }\right]}{E_{R}\left[{\tilde{\gamma }}^{\prime }\;\cdot \;R\;\cdot \;\tilde{\gamma }\right]}\\ \;=\frac{{\tilde{\gamma }}^{\prime }\;\cdot \;E_{R}\left[R^{2}\right]\;\cdot \;\tilde{\gamma }}{{\tilde{\gamma }}^{\prime }\;\cdot \;E_{R}[R]\;\cdot \;\tilde{\gamma }}\\ \;=\frac{{\tilde{\gamma }}^{\prime }\;\cdot \;E_{R}\left[R^{2}\right]\;\cdot \;\tilde{\gamma }}{{\tilde{\gamma }}^{\prime }\;\cdot \;\Sigma \;\cdot \;\tilde{\gamma }}\\ \;=\frac{{\tilde{\gamma }}^{\prime }\;\cdot \;(\Sigma^{2}\;+\;\frac{\Sigma^{2}+tr(\Sigma )\Sigma }{N})\;\cdot \;\tilde{\gamma }}{{\tilde{\gamma }}^{\prime }\;\cdot \;\Sigma \;\cdot \;\tilde{\gamma }},\;\text{see}\;45\\ \;\approx \frac{{\tilde{\gamma }}^{\prime }\;\cdot \;(\Sigma^{2}\;+\;\frac{M}{N}\Sigma )\;\cdot \;\tilde{\gamma }}{{\tilde{\gamma }}^{\prime }\;\cdot \;\Sigma \;\cdot \;\tilde{\gamma }}\\ \;=\frac{{\tilde{\gamma }}^{\prime }\;\cdot \;\Sigma^{2}\;\cdot \;\tilde{\gamma }}{{\tilde{\gamma }}^{\prime }\;\cdot \;\Sigma \;\cdot \;\tilde{\gamma }}+\frac{M}{N} \end{array}$$

From equations (38) and (39), we have

$$\begin{array}{l} \Phi_{R}\;\approx \frac{1}{M}\;\cdot \;\left(\frac{{\tilde{\gamma }}^{\prime }\;\cdot \;\Sigma^{2}\;\cdot \;\tilde{\gamma }}{{\tilde{\gamma }}^{\prime }\;\cdot \;\Sigma \;\cdot \;\tilde{\gamma }}+\frac{M}{N}\right)-\frac{1}{N}\\ \;=\frac{1}{M}\;\cdot \;\left(\frac{{\tilde{\gamma }}^{\prime }\;\cdot \;\Sigma^{2}\;\cdot \;\tilde{\gamma }}{{\tilde{\gamma }}^{\prime }\;\cdot \;\Sigma \;\cdot \;\tilde{\gamma }}\right) \end{array}$$

This motivates the following definition of Φ

(40) $$\Phi \;:=\frac{1}{M}\frac{{\tilde{\gamma }}^{\prime }\;\cdot \;\Sigma^{2}\;\cdot \;\tilde{\gamma }}{{\tilde{\gamma }}^{\prime }\;\cdot \;\Sigma \;\cdot \;\tilde{\gamma }}\;\;\text{general}\;\text{definition}$$

So, for **any** predictor $\tilde{\gamma }$,

(41) $$E\chi_{\text{twas}}^{2}\approx 1+\frac{Nh_{\delta }^{2}}{\left(1\;-\;\beta^{2}\tau^{2}\right)}\left(\frac{1}{M}\frac{{\tilde{\gamma }}^{\prime }\;\cdot \;\Sigma^{2}\;\cdot \;\tilde{\gamma }}{{\tilde{\gamma }}^{\prime }\;\cdot \;\Sigma \;\cdot \;\tilde{\gamma }}\right)+\frac{N\beta^{2}\tau^{2}}{1\;-\;\beta^{2}\tau^{2}}$$

We note that the result on the scenario when variants are independent (equation 27) can be seen as a special case of equation 41.

**Proof that** 1/M≤Φ

Recall

$$\Phi =\frac{1}{M}\frac{{\tilde{\gamma }}^{\prime }\;\cdot \;\Sigma^{2}\;\cdot \;\tilde{\gamma }}{{\tilde{\gamma }}^{\prime }\;\cdot \;\Sigma \;\cdot \;\tilde{\gamma }}$$

We will use (14)

$$1/\tau^{2}={\tilde{\gamma }}^{\prime }\;\cdot \;R\;\cdot \;\tilde{\gamma }\approx {\tilde{\gamma }}^{\prime }\;\cdot \;\Sigma \;\cdot \;\tilde{\gamma },$$

To find an extreme of Φ we will find an extreme of

$${\tilde{\gamma }}^{\prime }\;\cdot \;\Sigma^{2}\;\cdot \;\tilde{\gamma }$$

with the condition that

$$1/\tau^{2}={\tilde{\gamma }}^{\prime }\;\cdot \;\Sigma \;\cdot \;\tilde{\gamma ,}$$

using the Lagrange multiplier approach, i.e. we will find the extreme of the function

$$f(\lambda ,ℒ)={\tilde{\gamma }}^{\prime }\;\cdot \;\Sigma^{2}\;\cdot \;\tilde{\gamma }-ℒ\left({\tilde{\gamma }}^{\prime }\;\cdot \;\Sigma \;\cdot \;\tilde{\gamma }-1/\tau^{2}\right),$$

where ℒ is the Lagrange multiplier. We will also write the numerator in a more convenient form by using the eigenvalue decomposition of Σ. Since Σ is symmetric, it can be eigenvalue-decomposed

$$\Sigma =C\;\cdot \;\Lambda \;\cdot \;C^{\prime }$$

where C is the matrix of eigenvectors of Σ as columns and is orthonormal $C^{\prime }C=I.\;\Lambda$ is a diagonal matrix with the eigenvalues of Σ, $\lambda_{k}$’s.

$${\tilde{\gamma }}^{\prime }\;\cdot \;\Sigma \;\cdot \;\tilde{\gamma }\;={\tilde{\gamma }}^{\prime }\;\cdot \;C\;\cdot \;\Lambda \;\cdot \;C^{\prime }\;\cdot \;\tilde{\gamma }\;\;=\gamma_{C}^{\prime }\;\cdot \;\Lambda \;\cdot \;\gamma_{C}\;\;\text{with}\;\gamma_{C}:=C^{\prime }\;\cdot \;\tilde{\gamma }\;\;=\sum_{k}\;\gamma_{C,k}^{2}\lambda_{k}$$

Since the eigenvalues of $\Sigma^{2}$ are the squares of the eigenvalues of Σ, we have

$${\tilde{\gamma }}^{\prime }\;\cdot \;\Sigma^{2}\;\cdot \;\tilde{\gamma }\;={\tilde{\gamma }}^{\prime }\;\cdot \;C\;\cdot \;\Lambda^{2}\;\cdot \;C^{\prime }\;\cdot \;\tilde{\gamma }\;\;=\gamma_{C}^{\prime }\;\cdot \;\Lambda^{2}\;\cdot \;\gamma_{C}\;\;=\sum_{k}\;\gamma_{C,k}^{2}\lambda_{k}^{2}$$

Now, we rewrite the function f in terms of the eigenvalues of Σ

$$f(\lambda ,ℒ)=\sum_{k}\gamma_{C,k}^{2}\lambda_{k}^{2}-ℒ\left(\sum_{k}\;\gamma_{C,k}^{2}\lambda_{k}-\frac{1}{\tau^{2}}\right)$$

where ℒ is the Lagrange multiplier. The solution is obtained by setting the derivatives with respect to $\lambda_{k}$ and ℒ to 0.

$$2\;\gamma_{C,k}^{2}\lambda_{k}-ℒ\;\gamma_{C,k}^{2}=0\;\text{and}\;\;\sum_{k}\;\gamma_{C,k}^{2}\lambda_{k}-\frac{1}{\tau^{2}}=0$$

therefore

$$\lambda_{k}=\frac{ℒ}{2}\;\;\text{and}\;\;\sum_{k}\gamma_{C,k}^{2}\;\frac{ℒ}{2}=\frac{1}{\tau^{2}}$$

therefore

$$ℒ=\frac{2}{\tau^{2}\sum_{l}\;\gamma_{C,l}^{2}}⟹\lambda_{k}=\frac{1}{\tau^{2}\sum_{l}\;\gamma_{C,l}^{2}}\forall k$$

We can show that this is indeed a minimum of the function f by calculating the Hessian of the function and verifying that all the eigenvalues are positive definite. $f^{\prime \prime }=2\gamma_{C,k}^{2}>0$ for all k and cross derivatives are 0, so that the Hessian is diagonal so that its eigenvalues are $2\gamma_{C,k}^{2}>0$. Therefore, this solution corresponds to a minimum.

The minimum of the function corresponds to the case where all the eigenvalues are equal, therefore Σ is the identity matrix.

If we plug in the identity matrix in the equation for Φ, we get

$$\frac{1}{M}\leq \Phi$$

since $\frac{{\tilde{\gamma }}^{\prime }\cdot \Sigma^{2}\cdot \tilde{\gamma }}{{\tilde{\gamma }}^{\prime }\cdot \Sigma \cdot \tilde{\gamma }}=\frac{{\tilde{\gamma }}^{\prime }\tilde{\gamma }}{{\tilde{\gamma }}^{\prime }\tilde{\gamma }}=1$

**Proof that** Φ≤1

Recall

$$\Phi =\frac{1}{M}\frac{{\tilde{\gamma }}^{\prime }\;\cdot \;\Sigma^{2}\;\cdot \;\tilde{\gamma }}{{\tilde{\gamma }}^{\prime }\;\cdot \;\Sigma \;\cdot \;\tilde{\gamma }}$$

Using the eigenvalue decomposition of Φ, we can reexpress as:

$$\Phi =\frac{1}{M}\frac{\sum_{k}\;\gamma_{C,k}^{2}\lambda_{k}^{2}}{\sum_{k}\;\gamma_{C,k}^{2}\lambda_{k}}$$

Since the trace of Σ is $M,\sum_{k}\;\lambda_{k}=M$ and $\lambda \geq 0,\lambda_{k}\leq M$

Hence using $\lambda_{k}^{2}\leq \lambda_{k}M$:

$$\Phi \leq \frac{1}{M}\frac{\sum_{k}\;\gamma_{C,k}^{2}\lambda_{k}M}{\sum_{k}\;\gamma_{C,k}^{2}\lambda_{k}}=1$$

(42) Φ≤1

This upper bound is attained when the first eigenvalue equals M and all others are 0, i.e., all SNPs are perfectly correlated as seen below

$$\Phi =\frac{1}{M}\frac{\gamma_{C,1}^{2}M^{2}}{\gamma_{C,1}^{2}M}=1.$$

Since variance of $\tilde{T}$ has to be non zero, $\gamma_{C,1}^{2}$ is > 0.

**Decomposition of** $\sigma_{\epsilon }^{2}$

$$\begin{array}{l} \sigma_{\epsilon }^{2}\;=\sigma_{Y}^{2}-\sigma_{T}^{2}\beta^{2}-h_{\delta }^{2}\sigma_{Y}^{2}\\ \;=1-\beta^{2}-h_{\delta }^{2}\;\;∵\;\text{cross}\;\text{terms}\;=0\;\text{in}\;E\frac{Y^{\prime }Y}{N}\\ \;\approx 1-\beta^{2}\tau^{2}-h_{\delta }^{2}-\beta^{2}+\beta^{2}\tau^{2}\\ \;=1-\beta^{2}\tau^{2}-h_{\delta }^{2}-\beta^{2}\left(1-\tau^{2}\right) \end{array}$$

**Proof that** $E_{R}\left(R^{2}\right)=\Sigma^{2}+\frac{\Sigma^{2}+tr(\Sigma )\Sigma }{N}$

(Note that we are using $E_{R}$ to indicate that R is being integrated out, not conditioned on as in the rest of the derivation. Here we are not taking expectation with respect to R but using the fact that R and its quadratic forms are converging to their expected values.)

From Theorem 3.1 in (Haff, 1979), for a random variable $S\sim W_{M}(\Sigma ,N)$,

$$cov\left(S_{ij},S_{kl}\right)=N\;\cdot \;\left(\Sigma_{ik}\Sigma_{jl}+\Sigma_{il}\Sigma_{jk}\right)$$

Recall that $N\;\cdot \;R\sim W_{M}(\Sigma ,N)$ so $R=\frac{S}{N}$.

(43) $${\left[{\text{E}}_{R}\left(R^{2}\right)\right]}_{ij}={\text{E}}_{R}\left(R_{i}^{\prime }R_{j}\right)\;\;={\text{E}}_{R}\left(\sum_{k}R_{ki}R_{kj}\right)\;\;=\sum_{k}{\text{E}}_{R}\left(R_{ki}R_{kj}\right)$$

(44) $$E_{R}\left(R_{ki}R_{kj}\right)\;=E_{R}\left(R_{ki}\right)E_{R}\left(R_{kj}\right)+cov\left(R_{ki},R_{kj}\right)\;\;=\Sigma_{ki}\Sigma_{kj}+\frac{1}{N^{2}}\;\cdot \;N\;\cdot \;\left(\Sigma_{kk}\Sigma_{ij}+\Sigma_{ki}\Sigma_{kj}\right)\;\;=\Sigma_{ki}\Sigma_{kj}+\frac{\Sigma_{kk}\Sigma_{ij}\;+\;\Sigma_{ki}\Sigma_{kj}}{N}$$

(45) $$∴{\text{E}}_{R}\left(R^{2}\right)=\Sigma^{2}+\frac{\Sigma^{2}\;+\;\mathrm{tr}(\Sigma )\Sigma }{N},$$

from (43) and (44)

### Estimation of Φ for genes, metabolites, and brain features.

We downloaded prediction weights for gene expression in whole blood, metabolites in plasma, and brain features from the PredictDB website (Barbeira et al., 2021; Liang et al., 2021). We used the weights for the 7,131 genes, 1,156 metabolites, and 308 brain features to calculate predicted values using genotype data from unrelated White British UK Biobank individuals. We used HapMap3 subset of SNPs. We removed SNPs and individuals with missingness greater than 1%. Batches of 1,000, 5,000,10,000 individuals were selected for the analysis. After filtering, the sample sizes were 999, 4,992, and 9,979. We computed the genetically predicted gene expression and metabolite levels using the PrediXcan software (Barbeira et al., 2018). For brain features, we used the BrainXcan tool (Liang et al., 2022). We simulated the target trait Y using an infinitesimal model Y=X⋅δ+ϵ, where X was the genotype matrix from the UK Biobank and the M-dimensional vector δ and N-dimensional vector ϵ were sampled from independent standard normal distributions. All values were scaled to achieve the desired heritability. We calculated $E\chi^{2}$ for each gene, metabolite, and brain feature for a range of values of $h_{\delta }^{2}$ and N. Heritability ranged from 0.01 to 0.99. We calculated Φ for each gene, metabolite, and brain feature as the slope of the linear regression of $E\chi^{2}$ on the product of the sample size and heritability $Nh_{\delta }^{2}$. To borrow information across genes, we ran a mixed effects model where we allowed for random slope but constant intercept. We used the slope of the linear regression as an estimate of Φ for each gene, metabolite, and brain feature.

## Supplementary Material

Supplement 1
