# Supplementary material for: On the problem of inflation in transcriptome-wide association studies
Source: bioRxiv. 2023 Oct 20:2023.10.17.562831. Preprint. [Version 1] doi: 10.1101/2023.10.17.562831 (PMC10614931; doi:10.1101/2023.10.17.562831)
Supplement: Supplementary file 1 [file NIHPP2023.10.17.562831v1-supplement-1.pdf]

bioRxiv preprint doi: https://doi.org/10.1101/2023.10.17.562831; this version posted October 20, 2023. The copyright holder for this preprint (which was not certified by peer review) is the author/funder, who has granted bioRxiv a license to display the preprint in perpetuity. It is made available under aCC-BY 4.0 International license.

Supplementary Material

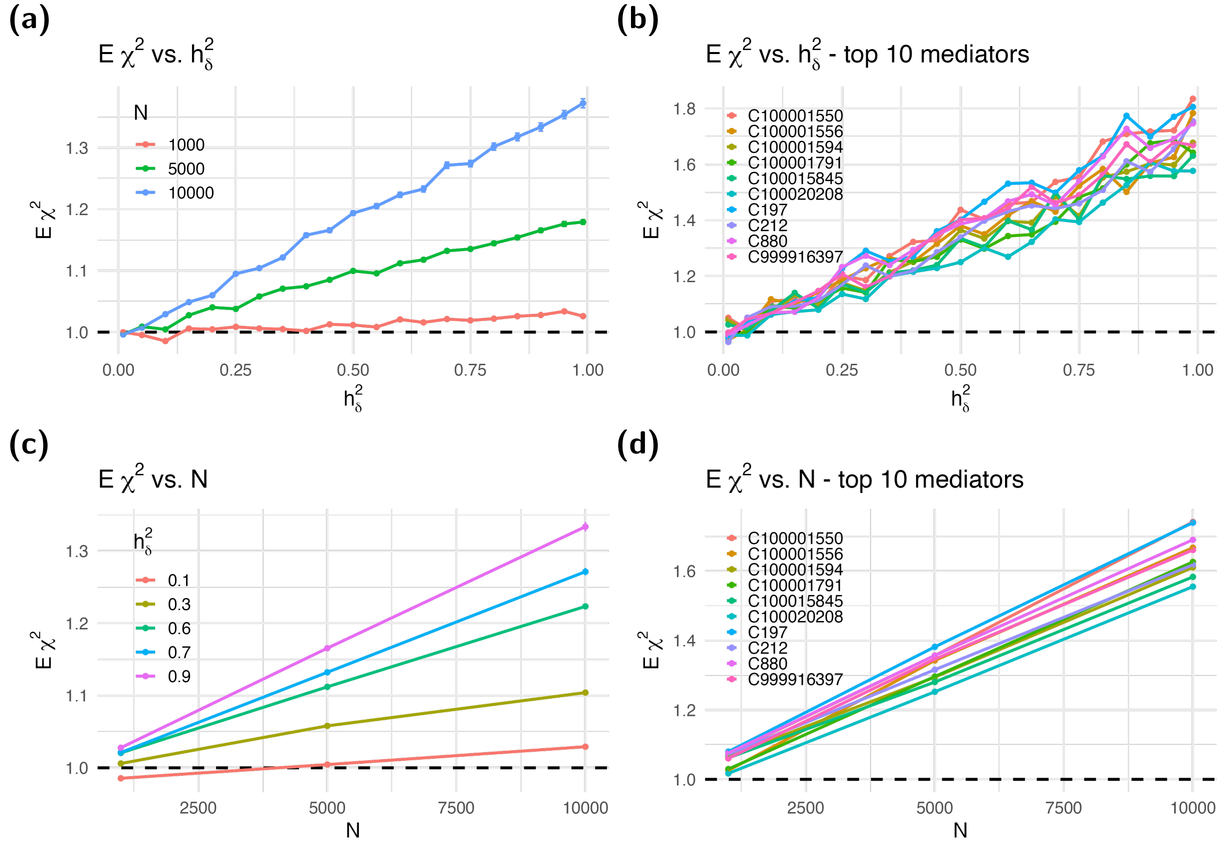

Figure S7: Inflation in MetaboXcan. Expected χ^2 statistics in TWAS calculated with simulated target traits with different heritability values of the target trait h^2_δ and sample sizes N. Estimated χ^2 is calculated averaging over 1000 simulated Y for a given metabolite, heritability, and sample size. **a** shows the average Eχ^2 over 1,156 metabolites χ^2 vs h^2_δ. **b)** shows the Eχ^2 for each of the top 20 most inflated metabolites vs h2. **c)** shows the average Eχ^2 over 1,156 metabolites vs N. **d)** shows the Eχ^2 for each metabolites vs N.

bioRxiv preprint doi: https://doi.org/10.1101/2023.10.17.562831; this version posted October 20, 2023. The copyright holder for this preprint (which was not certified by peer review) is the author/funder, who has granted bioRxiv a license to display the preprint in perpetuity. It is made available under aCC-BY 4.0 International license.

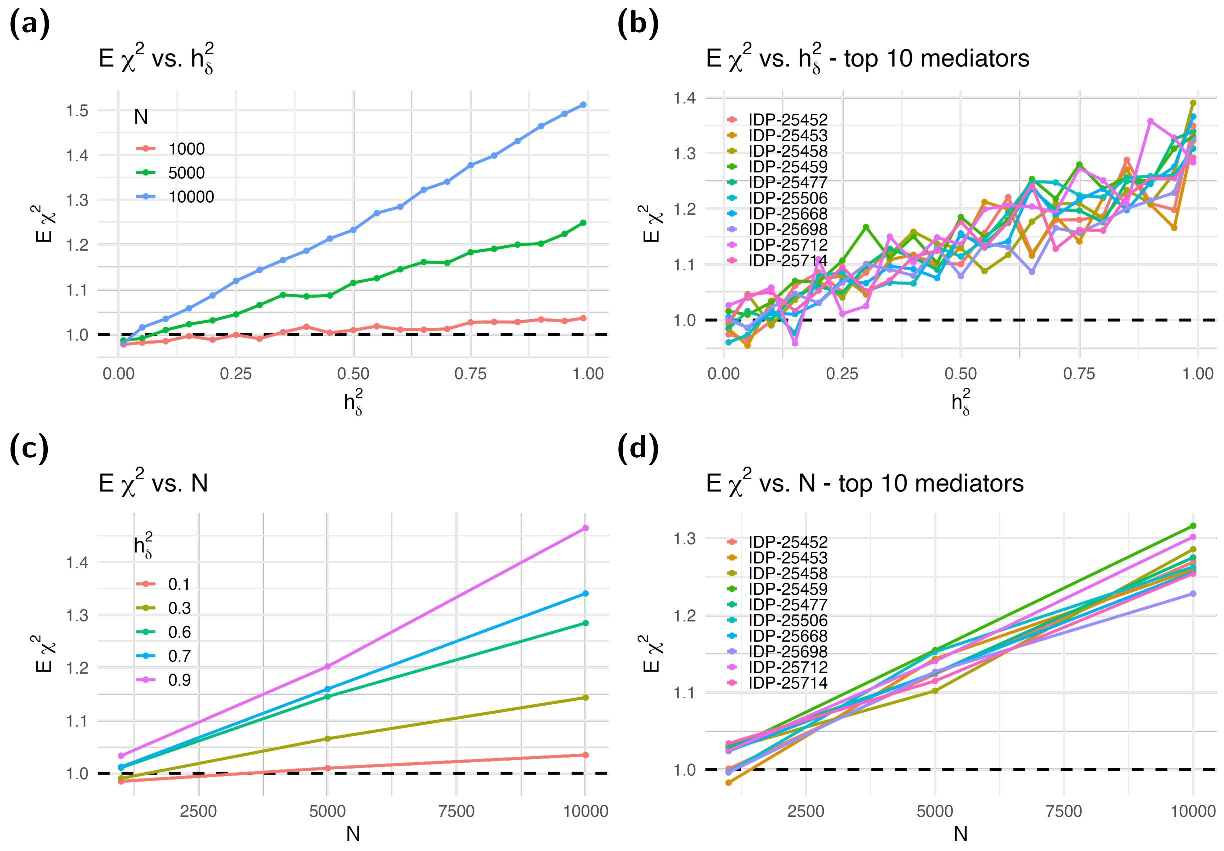

Figure S8: Inflation in BrainXcan. Expected χ^2 statistics in TWAS calculated with simulated target traits with different heritability values of the target trait h^2_δ and sample sizes N. Estimated χ^2 is calculated averaging over 1000 simulated Y for a given brain feature, heritability, and sample size. **a** shows the average $\mathrm{E}\chi^2$ over 308 brain features χ^2 vs h^2_δ. **b)** shows the $\mathrm{E}\chi^2$ for each of the top 20 most inflated brain features vs h2. **c)** shows the average $\mathrm{E}\chi^2$ over 308 brain features vs N. **d)** shows the $\mathrm{E}\chi^2$ for each brain feature vs N.
